# Enhancing adherence for total body skin examination in post-surgical veterans: an interventional study at an urban Veterans Affairs center

**DOI:** 10.1186/s40779-024-00552-5

**Published:** 2024-07-29

**Authors:** Vignesh Ramachandran, Efe Kakpovbia, Michelle C. Juarez, Neil Jairath, Andjela Nemcevic, Christine C. Akoh, Ian M. Ahearn, Ian W. Tattersall, Nayoung Lee, Jo-Ann M. Latkowski, John G. Zampella

**Affiliations:** 1 The Ronald O. Perelman Department of Dermatology, New York University, New York, NY 10016 USA; 2 Veteran Affairs New York Harbor Health Care, New York, NY 10010 USA

**Keywords:** Skin cancer screening, Quality improvement, Total body skin check, Veteran population

Dear Editor,

The Veterans Health Administration (VHA) plays a crucial role in the U.S. healthcare system, particularly for a population with a high prevalence of skin cancer [1]. Timely total body skin examination (TBSE) is vital for managing cutaneous malignancies [2]. Despite National Comprehensive Cancer Network (NCCN) guidelines advocating regular TBSE post-diagnosis [3], adherence remains suboptimal. We conducted an Institutional Board Review-exempt quality improvement interventional study aimed at increasing TBSE follow-up rates among veterans treated for skin cancer at the Manhattan Veterans Affairs Medical Center. Herein, we present an update to earlier proof-of-concept study data [4], now with statistically significant and applicable findings.

Our study employed a comprehensive quality improvement (QI) design with a single intervention, enabling direct comparison of post-intervention outcomes. As a QI project, this study was exempt from Institutional Board Review upon review. Patients who underwent standard excision (SE) or Mohs micrographic surgery (MMS) for skin cancer between July 2021 and August 2022 were identified. Recommended TBSE dates were obtained from electronic medical records using NCCN guidelines and the date of biopsy diagnosis. The intervention involved a targeted reminder phone call from a resident physician (VR, EK, MCJ, NJ, AN, CCA) 3 − 6 weeks before their scheduled TBSE to remind patients of a) their last type of skin cancer diagnosis; b) the date of the scheduled TBSE; c) the importance of follow-up given recent history of skin cancer. These findings were compared with retrospective control data from our VHA center (July 2018 to November 2019), where patients received standard appointment reminders in the form of mailed letters from our clinic. Statistical analysis was performed using Chi-squared tests, with statistical significance set at *P* < 0.05. Our all-comers’ design and uniform application of the intervention with a predetermined start date helped mitigate selection bias. In our dermatology clinic, separate appointments are made for general/medical dermatology visits, and TBSE is denoted as “health maintenance” problem visits in our documentation, thus eliminating this potential confounding factor.

The intervention group comprised 130 patients, while the control group consisted of 83 patients. All participants were proficient in English. Within the intervention group, 40% had squamous cell carcinoma (SCC), 47% had basal cell carcinoma (BCC), 8% had melanoma in situ (MIS), and 3% had basosquamous carcinoma. In contrast, the control group exhibited a distribution of 58% SCC, 35% BCC, and 7% MIS. Staging data was not collected due to the typical absence of staging for these skin cancers in our clinical dermatologic practice. The total time spent implementing the phone call intervention was approximately 6.5 h. A contingency table is represented in Table 1.

**Table 1:** Proportion of patients who appeared for total body skin examination (TBSE) after surgery

Types	Pre-intervention [*n* (%)]	Post-intervention [*n* (%)]	*χ*^2^	*P-*value
All Patients			11.72	< 0.001
Returned for TBSE	50 (60.2)	106 (81.5)		
Did not return for TBSE	33 (39.8)	24 (18.5)		
Squamous cell carcinoma (SCC)			5.16	0.023
Returned for TBSE	30 (62.5)	43 (82.7)		
Did not return for TBSE	18 (37.5)	9 (17.3)		
Basal cell carcinoma (BCC)			8.35	0.003
Returned for TBSE	16 (55.2)	51 (83.6)		
Did not return for TBSE	13 (44.8)	10 (16.4)		
Melanoma in situ (MIS)			0.02	0.889
Returned for TBSE	4 (66.7)	7 (70.0)		
Did not return for TBSE	2 (33.3)	3 (30.0)		

The intervention group demonstrated significantly higher adherence to TBSE compared with the control group (81.5% vs. 60.2%, *P* < 0.001). When stratified by cancer type, the intervention group showed increased compliance with TBSE following BCC and SCC diagnosis compared with the control group (SCC: 82.7% vs. 62.5%, *P* = 0.0231; BCC: 83.6% vs. 55.2%, *P* = 0.003). However, there was no significant change in MIS compliance (70.0% vs. 66.7%, *P* = 0.889) (Table 1).

The limitations of this study include the inability to assess qualitative factors such as patient knowledge, behaviors, and modifiable risk factors, the absence of data regarding previous skin cancer diagnosis and its impact on a patient’s willingness to adhere to follow-up, a predominantly homogenous patient population, as well as non-standardization scripts for phone calls. Although our sample was largely homogenous (comprising elderly white males), we were unable to gather demographic data which constitutes an additional constraint in assessing the potential impact of demographic variables on adherence. Moreover, socioeconomic status and underlying health may have affected our findings. These areas warrant future research and intervention for our group. While confident in the adaptability of our intervention at other VA sites due to serving a bustling metropolitan area with a strong demand for services, we acknowledge that its implementation was facilitated by the scale of our residency program. Smaller programs could explore automated systems, an option under consideration for subsequent studies.

Overall, the implementation of reminder phone calls led to a significant improvement in follow-up rates for TBSE, particularly among patients treated for BCC and SCC, as well as those undergoing MMS. This study extends previous research highlighting the impact of reminders on healthcare compliance, demonstrating enhanced adherence to scheduled TBSE, although the findings were not statistically significant [4].

Future directions involve the integration of a standardized, automated system, conducting a comprehensive cost analysis, gathering additional demographic data, performing subgroup analyses, and examining the impact of language on patient perceptions. Furthermore, it is essential to assess how this intervention influences skin cancer detection during TBSE visits [5].

We encourage other VHA systems to identify and disseminate their strategies for improving the health of our veterans by reducing morbidity and mortality associated with delayed TBSE.

## Data Availability

The data that support the findings of this study are available from the corresponding author, VR, upon reasonable request.

## Abbreviations

BCC: Basal cell carcinoma
MIS: Melanoma in situ
MMS: Mohs micrographic surgery
SCC: Squamous cell carcinoma
NCCN: National Comprehensive Cancer Network
TBSE: Total body skin examination
VHA: Veterans Health Administration
